# Recurrent acute pancreatitis and massive hemorrhagic ascites secondary to a duodenal duplication in a child: a case report

**DOI:** 10.1186/1752-1947-7-70

**Published:** 2013-03-14

**Authors:** Min Yang, Ding-You Li, Yong-Mei Zeng, Pei-Yu Chen, Lan-Lan Geng, Si-Tang Gong

**Affiliations:** 1 Department of Gastroenterology, Guangzhou Women and Children’s Medical Center, Guangzhou, China; 2 Department of Pediatrics, Section of Gastroenterology, University of Missouri-Kansas City, Children’s Mercy Hospital, Kansas City, Missouri, United States of America

**Keywords:** Acute pancreatitis, Child, Duodenal duplication, Hemorrhagic ascites

## Abstract

**Introduction:**

Duodenal duplication is a rare congenital malformation and has been reported as a rare cause of recurrent acute pancreatitis. Hemorrhagic ascites has been reported in only one case of duodenal duplication.

**Case presentation:**

An 11-year-old Chinese girl presented with abdominal pain, hematemesis and dark stools. On admission, an abdominal examination revealed a moderately distended abdomen with diffuse tenderness. Biochemical investigations showed increased serum levels of amylase, lipase, and urine amylase. An abdominal computed tomography scan and magnetic resonance imaging scan revealed an enlarged and heterogeneous pancreas with poorly delineated borders. There was a cystic lesion measuring 25mm × 48mm × 28mm, located between the descending portion of her duodenum and the head of her pancreas. There were massive effusion signals in her abdominal cavity. An exploratory laparotomy was performed. A tubular cyst measuring 32mm × 52mm × 30mm was found in the second part of the duodenum, next to the head of her pancreas. The anterior wall of the duplication cyst was resected and anastomosis of the remaining cyst to the duodenum was performed for drainage. Histopathological examination of the excised cyst wall showed duodenal mucosa, submucosa and muscle coats, indicative of a duodenal duplication.

**Conclusions:**

It is important to be aware of duodenal duplication when evaluating a patient with recurrent acute pancreatitis accompanied by massive hemorrhagic ascites.

## Introduction

Duodenal duplication is a rare congenital malformation. It is usually diagnosed in infancy and childhood [1], with 60% of patients presenting at ages younger than 2 years old [2]. It can be cystic or tubular, communicating or non-communicating. It may result in several complications, such as obstruction, bleeding, perforation, and jaundice. It has been reported as a rare cause of recurrent acute pancreatitis [3]. Hemorrhagic ascites has been reported in only one case of duodenal duplication [4]. Here we report an extremely rare case of a previously healthy 11-year-old girl with recurrent acute pancreatitis and massive hemorrhagic ascites, who was subsequently diagnosed with tubular and non-communicating duodenal duplication.

## Case presentation

An 11-year-old Chinese girl presented with 6-months history of epigastric abdominal pain, and 4 days of hematemesis and dark stools. Her past medical history was unremarkable. On admission, physical examination showed a moderately distressed patient with a temperature of 36.8°C, a respiratory rate of 20 breaths/minute, a blood pressure of 95/65mmHg and a pulse rate of 108 beats/minute. An abdominal examination revealed a moderately distended abdomen with diffuse tenderness. There was positive shifting dullness and diminished bowel sounds, but no abdominal wall rigidity. A complete blood count revealed a white blood cell count of 10.6×10^9^/L with 54.1% neutrophils, a hemoglobin level of 110g/L and hematocrit of 35.8%. Examination of stool parasites showed *Ascaris* eggs: 0 to 2/high power field. Biochemical investigations showed increased serum levels of amylase (1665U/L; normal 30U/L to 110U/L), lipase (287U/L; normal 5.6U/L to 51.3U/L) and urine amylase (6059U/L; normal 32U/L to 641U/L). Liver transaminases, total bilirubin, alkaline phosphatase, glucose, electrolytes and renal function tests were within the normal ranges. Diagnostic paracentesis revealed dark brown-colored fluid containing a great quantity of red blood cells and white blood cells (243×10^9^/L). The fluid amylase was 15,240U/L. The result of her Rivalta test was positive.

**Figure 1:**
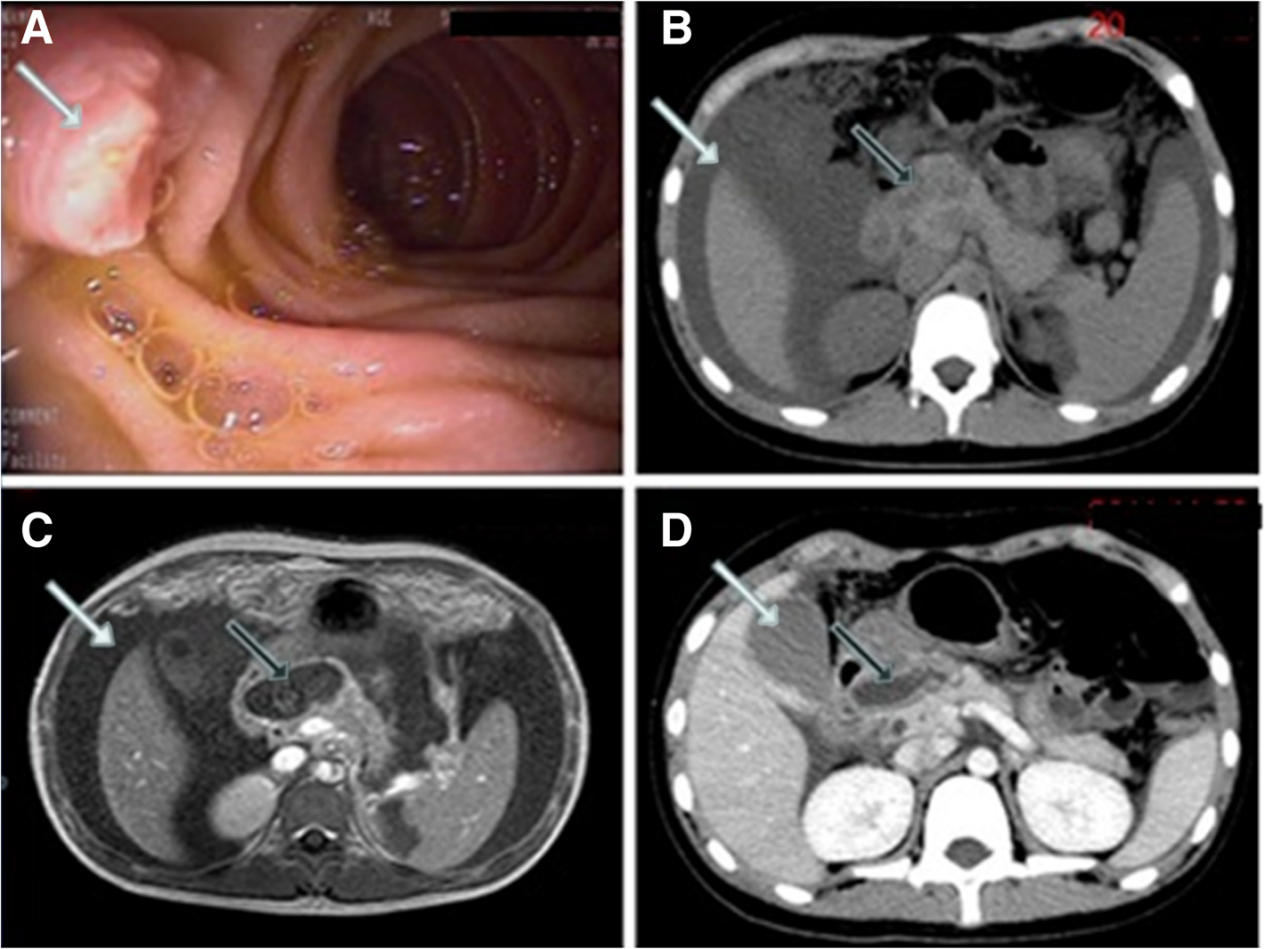
**(A) Upper gastrointestinal endoscopy: the pancreatic papilla was swollen (white arrow).** (**B**) Abdominal computed tomography (CT): A cystic lesion located between the descending portion of the duodenum and the head of pancreas (black arrow) and massive effusion signals in the abdominal cavity (white arrow). (**C**) Abdominal magnetic resonance imaging: A thick-walled cystic lesion with low signal intensity between the second part of the duodenum and the head of pancreas (black arrow), and massive effusion signals in the abdominal cavity (white arrow). (**D**) Abdominal CT: A well-delineated cystic lesion located between the descending portion of the duodenum and the head of pancreas. This lesion was not enhanced by the administration of an intravenous contrast: gadolinium (black arrow). The gallbladder was enlarged with high density in the bottom of the gallbladder (white arrow).

Abdominal ultrasonography revealed a slightly enlarged liver and heterogeneous echogenicity of her pancreas. Her main pancreatic duct was dilated. Her gallbladder, biliary duct system and spleen were normal. Severe ascites was detected. An upper gastrointestinal endoscopy revealed a swollen pancreatic papilla. There was no diverticulum or polypoid lesion protruding into the lumen of the second part of her duodenum (Figure 1A). An abdominal computed tomography (CT) revealed an enlarged and heterogeneous pancreas with poorly delineated borders. There was a cystic lesion measuring 25mm × 48mm × 28mm, located between the descending portion of her duodenum and the head of the pancreas. There were massive effusion signals in the abdominal cavity. The gallbladder and bile duct were normal (Figure 1B). The patient was diagnosed with acute pancreatitis accompanied by a possible pancreatic pseudocyst. Fasting, intravenous fluid therapy, somatostatin, albendazole and antibiotics were initiated. One week later, an abdominal magnetic resonance imaging (MRI) scan confirmed a heterogeneous pancreas with poorly delineated borders, and a cystic lesion between the second part of the duodenum and the head of the pancreas, measuring 25mm × 46mm × 25mm. Again, massive effusion signals were seen in the abdominal cavity (Figure 1C). Magnetic resonance cholangiopancreatography demonstrated a normal biliary tree. The gallbladder was enlarged with smooth walls. The cystic lesion was thought to be an edematous pancreatic pseudocyst. At this point, enteral nutrition therapy via a nasojejunal tube was initiated. During the hospitalization, enteral nutrition was attempted twice and both times the patient developed acute pancreatitis with massive hemorrhagic ascites that were clinically and laboratory confirmed. The patient was treated conservatively with complete bowel rest and administration of parenteral nutrition, somatostatin and antibiotics. After the third episode of acute pancreatitis, an abdominal CT was repeated and showed a well-delineated cystic lesion located between the descending portion of the duodenum and the head of the pancreas. The lesion was not enhanced by intravenous contrast. The gallbladder was enlarged with high density in the bottom portion. Massive effusion signals had disappeared in the abdominal cavity (Figure 1D).

An exploratory laparotomy was performed. A tubular cyst measuring 32mm × 52mm × 30mm was found in the second part of the duodenum, next to the head of the pancreas. Its surface was adjoined with the pancreatic surface. The cyst was non-communicating with duodenum, stomach and pancreaticobiliary system. The anterior wall of the duplication cyst was resected and anastomosis of the remaining cyst to duodenum was performed for drainage. During surgery, biopsies were obtained and the remnant cyst wall was thoroughly examined to exclude any abnormalities or ectopic tissues. The gallbladder appeared enlarged and the wall was edematous. The gallbladder and bile duct were full of sand-like stones. Histopathological examination of the excised cyst wall showed duodenal mucosa, submucosa and muscle coats, indicative of a duodenal duplication. There was no evidence of malignancy or dysplasia. The postoperative course was uneventful and 15 days later the patient was discharged home. At one year follow-up visit, she remained asymptomatic with normal serum amylase and lipase. There were no clinical signs of ascites.

## Discussion

Duodenal duplication is a rare congenital disorder and accounts for only 5% to 7% of all gastrointestinal duplications [1]. They are mostly limited to the first or second part of the duodenum usually adjacent to the pancreatic surface sharing a common wall and blood supply with the duodenum. The common clinical presentation in children is vomiting, abdominal pain, or abdominal mass. Acute pancreatitis has been reported in a few cases as a rare complication [3] and hemorrhagic ascites in one case report [4]. To the best of our knowledge, this is the first reported case of combined recurrent acute pancreatitis and hemorrhagic ascites caused by duodenal duplication in a child.

There are several possible mechanisms for pancreatitis to develop from a duplication cyst [5]. In this case, the duodenal duplication cyst most probably caused mechanical obstruction of the pancreatic duct and subsequent acute pancreatitis, given the fact that the initial ultrasonography confirmed pancreatic duct dilatation. The non-communicating cyst may produce viscid mucous secretions and mucosal inflammation which may cause distention of the cyst and obstruction of the pancreatic duct [6]. We do not think that gallstones contributed to acute pancreatitis in our case, because no gallstones were identified on multiple imaging studies. The sand-like stones found in the gallbladder were probably due to long-term fasting and somatostatin. *Ascaris* infestation is probably an incidental finding rather than a cause for acute pancreatitis, because thorough examination during surgery did not find any adult *Ascaris* in the pancreaticobiliary system. We speculate that hemorrhagic ascites may have been the result of mucosal inflammation, necrosis and micro-perforation of the duplication cyst. Acute necrotizing pancreatitis can cause ascites but would be an unlikely cause of the hemorrhagic ascites in our case because neither imaging studies nor exploratory laparotomy revealed a necrotic pancreas.

Multiple reports found that both CT imaging and MRI can adequately identify a duodenal duplication cyst [3,6]. However, due to the rarity of duodenal duplication cyst and lack of clinical experiences, more common lesions such as pancreatic pseudocyst are often suspected. In fact, duodenal duplication cyst was reported to mimic pancreatic cyst in a patient with acute pancreatitis [7]. Kawahara *et al*. [8] found 25 children with duodenal duplication presenting as pancreatitis. In 10 cases, the duodenal duplications were not in continuity with the duodenum and the locations of the duplications were variable. Six were within the pancreas: four in the pancreatic head, one in the aberrant lobe connected with the main pancreatic lobe, and one in the pancreatic tail. Of interest, a pancreatic pseudocyst was associated with the duodenal duplications in eight of the 25 reported cases. Abdominal CT and abdominal ultrasound were the most frequently used diagnostic tests in the reported cases [8]. In our case, both CT and MRI images showed a well-delineated cystic lesion located between the descending portion of the duodenum and the head of pancreas. However, duodenal duplication was never suspected, probably due to the severity of pancreatitis and accompanying hemorrhagic ascites. The upper gastrointestinal endoscopy did not help in our case because the cyst was non-communicating with either duodenum or stomach. An abdominal ultrasonography failed to reveal any cystic lesion in our case.

The surgical intervention for duodenal duplication cyst includes complete or partial surgical resection of the cyst. The location of the cysts in relation to the duodenum, especially to the ampulla, is important to determine the treatment strategy [3,6,8]. Alternatively, duodenum duplication can be safely and effectively treated by different endoscopic interventions [3,9]. Antaki *et al*. [9] reported that eight patients were treated with endoscopic incision and marsupialization for symptomatic intraluminal duodenal duplication cysts. All patients remained asymptomatic at a median follow-up of 7.3 years. Although a duodenal duplication cyst is considered a benign clinical entity, three cases with development of malignancy have been reported [10]. Therefore, a long-term follow-up would be necessary for patients who had undergone partial resection of a duodenal duplication cyst.

## Conclusions

We described a very rare case of a child who was diagnosed with massive hemorrhagic ascites secondary to a non-communicating duodenal duplication cyst, which may mimic pancreatic pseudocyst in a patient with acute pancreatitis. Both CT imaging and MRI can adequately identify a duodenal duplication cyst. Our patient underwent a successful partial surgical resection. It is important to be aware of duodenal duplication when evaluating a patient with recurrent acute pancreatitis accompanied by massive hemorrhagic ascites.

## Consent

Written informed consent was obtained from the patient’s parents for publication of this case report and any accompanying images. A copy of the written consent is available for review by the Editor-in-Chief of this journal.

## Competing interests

The authors declare that they have no competing interests.

## Authors’ contributions

MY contributed to conception and design of the manuscript, clinical management of the patient, and drafted the manuscript. YZ, PC, and LG contributed to acquisition, analysis and interpretation of data, and drafting of the manuscript. DL and SG contributed to critical revision of the manuscript for important intellectual content. All authors read and approved the final manuscript.
